# Minimal residual disease in melanoma: circulating melanoma cells and predictive role of MCAM/MUC18/MelCAM/CD146

**DOI:** 10.1038/cddiscovery.2017.5

**Published:** 2017-03-06

**Authors:** Maria Cristina Rapanotti, Elena Campione, Giulia Spallone, Augusto Orlandi, Sergio Bernardini, Luca Bianchi

**Affiliations:** 1 Department of Laboratory Medicine, University of 'Tor Vergata', Rome, Italy; 2 Department of Dermatology, University of 'Tor Vergata', Rome, Italy; 3 Department of Anatomic Pathology, University of ‘Tor Vergata’, Rome, Italy

## Abstract

Circulating tumour cells (CTCs), identified in numerous cancers including melanoma, are unquestionably considered valuable and useful as diagnostic and prognostic markers. They can be detected at all melanoma stages and may persist long after treatment. A crucial step in metastatic processes is the intravascular invasion of neoplastic cells as circulating melanoma cells (CMCs). Only a small percentage of these released cells are efficient and capable of colonizing with a strong metastatic potential. CMCs' ability to survive in circulation express a variety of genes with continuous changes of signal pathways and proteins to escape immune surveillance. This makes it difficult to detect them; therefore, specific isolation, enrichment and characterization of CMC population could be useful to monitor disease status and patient clinical outcome. Overall and disease-free survival have been correlated with the presence of CMCs. Specific melanoma antigens, in particular MCAM (MUC18/MelCAM/CD146), could be a potentially useful tool to isolate CMCs as well as be a prognostic, predictive biomarker. These are the areas reviewed in the article.

## Facts:

- Circulating tumour cells (CTCs) are detectable at all stages of disease and persist long after treatment.
- CTCs are detectable, even when patients are considered disease-free, using antibodies against specific cell surface markers.
- Circulating melanoma cells (CMCs) are thought to be responsible for metastatic progression, making it especially lethal.
- Certain CMCs could be useful to monitor the disease status.
- Melanoma does not express the classical epithelial cell surface markers, such as EpCAM, which has formed the basis of most CTC isolation strategies.
- Expression of melanoma cell-adhesion molecule MCAM (MUC18/MelCAM/CD146) is associated with an aggressive, invasive phenotype and its upregulation is strongly associated with disease progression.

## Open Questions:

- How can CMCs be isolated? Which genotype and phenotype do they express?
- Have all CMC phenotypes a metastatic potential? Which phenotype should be considered dangerous?
- As their detection in melanoma patients is useful to monitor the disease status, can CMCs have a prognostic value?
- Can the CMCs' enumeration and characterization determine and predict the overall and disease-free survival?
- Can MCAM (MUC18/MelCAM/CD146), as one of the most melanoma-specific cell-surface epitope, be regarded either as a useful tool to isolate CMCs or as a possible prognostic or predictive biomarker of melanoma progression?

## Circulating Tumour Cells

In cancer, the metastatic spreading of cells from a primary tumour to distant sites is mostly responsible for patient morbidity and mortality.^1^ Millions of cells are shed from primary tumour every day (approximately 4×10^6^ cells/g primary tumour)^2,3^ invading lymphatic and/or venous circulatory systems. Once they enter into the bloodstream, they become circulating tumour cell population (CTCs).

**Figure 1:**
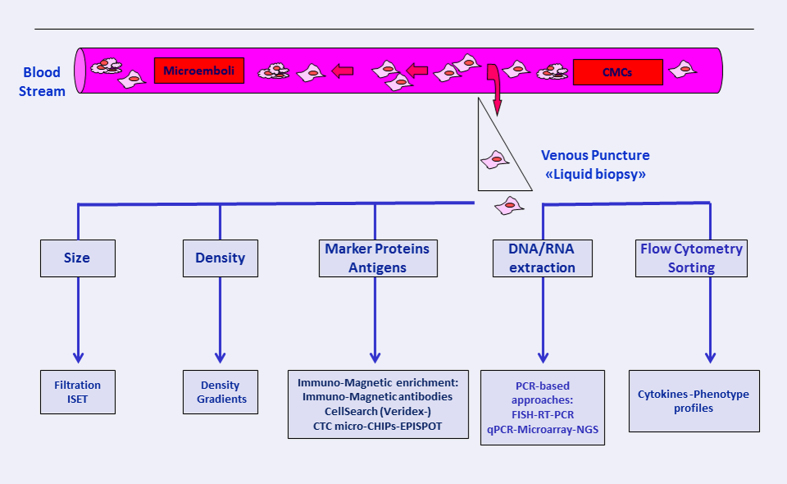
Schematic diagram of the main current methods for detection, isolation and enrichments of CTCs.

Fortunately, in cancer metastasis, only a small percentage of released cells are efficient and capable of colonizing as well as forming distant lesions, as survival can be limited by immune surveillance or haemodynamic forces.^4,5^ Experimental studies documented that only 0.01% of cancer cells injected into circulation form metastatic foci.^5,6^ It is not clear at which steps of the process the cells are lost, but the fact that metastasis can occur many years later after diagnosis and surgery indicates that most disseminated CTCs have been rapidly destroyed in the circulation or that they may remain apparently quiescent. Nevertheless, the presence of CTCs is a prerequisite step to establish distant metastases during the course of a given cancer and these cells are considered appropriately as the ‘leukaemic’ phase of solid tumour.^7^ CTCs acquire activating changes that lead to their extravasation into the surrounding tissue, degradation of basement membrane and extracellular matrix, capacity to migrate, adhere and propagate via the lymphatic and circulatory systems and establish new colonies at distant sites that will lead to metastatic disease.^7^ In one cubic centimetre of tissue, it has been established there are 10^9^ cells that rapidly can reach 10^10^–10^11^ cells before becoming clinically detectable.^8^ The ability to measure CTCs represents a potential powerful method to monitor patients with known malignancies who have a minimal morbidity. Despite metastasis is a relatively inefficient process, researchers and clinicians believe that CTCs can be effectively used to screen early-stage cancers. Detection of these CTC micrometastases seems to be useful for patient stratification groups with low or high risk for metastatic disease^9–16^ and as an independent prognostic marker in a variety of metastatic cancer.^17–23^ CTCs number, prior to initiation, during and after therapy, has been shown to be indicative of the length of progression-free survival (PFS) and overall survival (OS);^17,19,21,23–25^ moreover, its monitoring during and after therapy showed a correlation with the clinical course of the disease and the response to therapy.^17,22,26,27^ Predictive value for survival, based on CTC enumeration has been shown to be superior to standard monitoring tests.^18,28,29^ Detection, genotyping, phenotyping and molecular characterization of CTCs and more recently of cell-free circulating nucleic acids (in particular, circulating tumour DNA, ctDNA) have introduced the ‘new concept’ of ‘liquid biopsy’. ctDNA fragments mainly originate from apoptotic or necrotic tumour cells, which discharge their DNA into the blood circulation in several malignancies. They have proven to be of prognostic value in several cancers and have been detected with a sensitivity ranging from 26 to 100% analysing different genetic markers.^30^ Although molecular targets were initially detected in nucleic acid samples extracted from tumour tissue, the detection of nucleic acids in circulating blood has allowed alternative sample source for the identification of genomic alterations. Biopsy of overt metastases is an invasive procedure limited to certain locations and not easily acceptable in the clinic; 'liquid biopsy’ can be conducted serially and might allow real-time monitoring of cancer therapies in individual patients. Both CTCs and ctDNA are interesting complementary technologies that can be used in parallel in future trials assessing new drugs or drug combinations and may provide clinically relevant information that could rapidly revolutionize oncology practice.^31^ We have just started understanding the prognostic information inherent to ‘liquid biopsies’, while the methods by which CTCs and genetic markers should be monitored are still evolving and require close attention (Figure 1).^31^

### Circulating melanoma cells

Human melanoma is the most malignant skin cancer with rapidly increasing incidence in industrialized countries worldwide. Although melanoma can be treated by surgical resection, metastatic melanoma is one of the most aggressive and drug-resistant neoplasm with poor OS.^32,33^ Current prognostic techniques are inadequate for disease management as many patients, considered clinically disease-free following primary tumour resection, later develop metastases. The 10-year survival rate for non-metastatic patients ranges from 39 to 93%, depending on primary tumour thickness, mitotic rate and presence of ulceration. There is no measure of residual disease in early-stage patients postsurgery; thus those requiring treatment cannot be identified. More sensitive procedures need to be developed in order to stage patients in a more accurate manner.^34–36^

**Figure 2:**
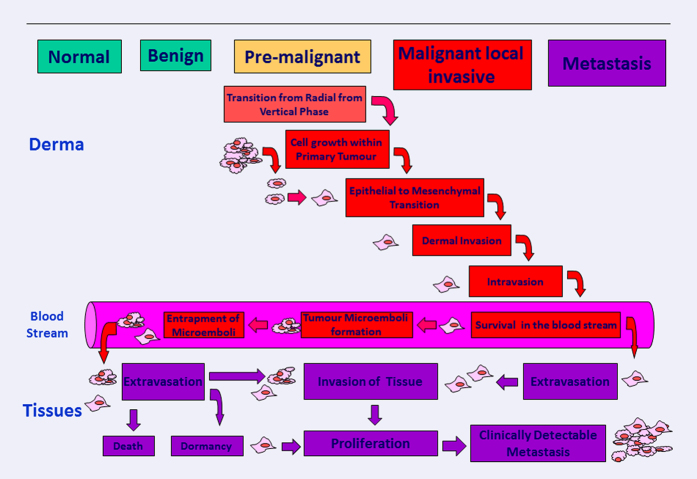
Path of metastasizing melanoma: from the detachment from primary tumour through the establishment of a metastatic tumour from CMCs.

It is now recognized that dissemination and implantation at distant sites of melanoma cells is a complex multi-step process influenced by both host and tumour characteristics.^8,35,37,38^ When cancer cells detach from the primary tumour, they can enter into blood or lymphatic system actively or passively. Passive intravasation is due to the detachment of CMCs from primary tumour as a consequence of increased haemodynamic flow.^39–43^ Recent biological data suggest that the active dislodgment of CMCs from the primary tumour is due to the acquisition of distinct modified phenotype and genotype that confer aggressiveness and metastatic efficiency.^39^ However, CMCs heterogeneity has a crucial role by allowing these cells to survive in the circulation and metastasize.^39^ Melanoma progression comprises transition from radial to vertical growth phase, epithelial-to-mesenchymal transition (EMT), alterations in cell adhesion properties and suppression of apoptosis.^44,45^ Precondition for metastasis is that surviving CMCs are able to adhere to endothelial surfaces and subsequently migrate into adjacent parenchyma in the target organ. More in detail, the key steps are: loss of adhesion, dermal invasion, migration from primary site, intravasation and subsequent survival in circulation, migration into distant tissues and subsequent proliferation, and enhancement of angiogenesis at the new colonized sites (Figure 2).^33,39,46^

E-CADH mediates homophilic cell–cell adhesion and has a crucial role in both epithelial cell–cell adhesion and in the maintenance of normal tissue architecture. Loss of E-CADH in normal melanocytes leads to the transition from benign lesions to invasive and metastatic cancer. It is considered as a tumour-suppressor gene acting as invasion-suppressor molecule: its loss permits and enhances invasion of adjacent normal tissues. Downregulation or complete shutdown of the E-CADH expression, mutation of the gene or other interference mechanisms involving the adherent junctions are strongly correlated with the loss of epithelial morphology and acquisition of metastatic potential.^47^ Once melanoma cells become invasive, they no longer express E-CADH but rather VE-CADH, (CDH5, Non-Epithelial Cadherin 5) or N-CADH (CDH2, Cadherin 2) both principally involved in EMT.^47,48^ Mel^39^ describes the process as follows: ‘the EMT process is used by migrating cells during embryonic development. It involves switching of polarized epithelial cells to contractile, motile mesenchymal progenitor cells, and is triggered by secretion of growth factors EGF (epithelial growth factor), FGF (fibroblast growth factor), and chemotactic/pro-migratory factors SF/HGF (hepatocyte growth factor) and chemokines from stromal fibroblasts and macrophages’.^39^

Because of EMT, melanocytic cells acquire the characteristics of their mesenchymal progenitor cells.^46,49^ Invasion is stimulated by increased activity of Wnt 5a and Notch pathways (increased expression of transcription factors Twist and Snail) and activation of PKC causing cytoskeletal changes that enhance cell motility.^49,50^ Twist, in particular, has an essential role in inhibiting E-CADH expression, inducing cell motility and contributing to metastasis by promoting EMT.^51–53^ In addition, E-CADH acts as an antiapoptotic factor enhancing cancer cell survival.^54^

Increasing evidence has suggested that phosphatase and tensin homolog (PTEN) is one of the powerful switches for the conversion between tumour suppressors and oncogenes. PTEN regulates a number of cellular processes, including cell growth, differentiation, cell death, proliferation, survival, motility, invasion and intracellular trafficking, through the phosphoinositide 3-kinase/protein kinase B/mammalian target of rapamycin (PI3K/AKT/mTOR) pathway. Furthermore, a number of studies have suggested that PTEN deletions may alter various functions of certain tumour-suppressor and oncogenic proteins. Alterations in this pathway, PTEN loss and/or AKT activation switches, mainly PTEN inactivation, have been associated with resistance to apoptosis and progression to tumorigenesis.^49,55–58^ Other proteins, involved in signalling pathways are enrolled in the evasion of apoptosis by CMCs, including a decrease in the expression of death receptors, increased expression of matrix metalloproteinases, increased secretion of growth factors and overexpression of antiapoptotic proteins (BCL-2, BCL-XL).^57^

It is renowned that such an aggressive tumour growth is strictly linked to the presence of blood vessels within and around tumour parenchyma^59,60^ as well as to the parallel dynamic expansion of the vasculature.^61–63^ The ability of tumour cells to recruit and maintain a ‘private’ vascular supply is achieved through several cellular processes involving interactions between tumour cells and their adjacent vascular endothelium, enhancing tumour growth.^33^ It is clear indeed that blood vessel formation process is complex and involves various interactions well exhaustively reviewed in literature.^64^ It is important to consider that cancer neovascularization must be triggered at a relatively early stage of tumour progression by a seemingly distinct event referred to as ‘the Angiogenic Switch’.^61^ This ‘Angiogenic Switch’ is a result of a change in balance between angiogenesis stimulators inhibitors and modulators present at the site of tumour growth.^61,65^ It seems to be influenced by genetic factors: more than 20 transforming oncogenes (that is, ras, myc, EGFR, HER-2), a large number of tumour-suppressor genes (that is, p53, PTEN) and epigenetic nature, such as hypoxia, inflammation and hormonal stimulation.^65–72^ Angiogenesis is the crucial event in melanoma metastatic progression and sustenance of cells within tumour.^64^

## Detection of CMCs

### Melanoma-associated antigen expression analysis

CMCs were studied for the first time in 1991 by detecting tyrosinase (Tyr-OH)—key enzyme of melanogenesis—in peripheral blood where the expression of this transcript is not normally expected.^73–79^

Various methods have been used to quantify and characterize CMCs in peripheral blood, without tumour cell separation, including indirect methods, such as reverse transcriptase-PCR (RT-PCR) and quantitative-RT PCR (qRT-PCR). Indirect methods are based on the assumption that, as melanocytes do not circulate, specific detection of melanocytic transcripts should correlate with CMCs.^39,80,81^ These cells are detectable in peripheral blood either soon after the surgical resection of primary tumours, regardless of their thickness, late-stage disease and even in clinically disease-free patients.^76,82^ These findings are confirmed by the percentage of positive cases for CMCs, ranging from 6 to 93% of the reports.^75,77,78,83–85^

RT-PCR is a variant of PCR. In RT-PCR, the RNA template is at first converted into a complementary DNA (cDNA) using a reverse transcriptase. cDNA is then used as a template to exponentially amplify target DNA sequences.^86^ Multiple markers RT-PCR assay has been established as the most reliable and sensitive approach to identify CMCs in peripheral blood or in draining lymph nodes of melanoma patients that express the putative transcripts of tumour-specific genes.^74,75,77,79,85,87–89^ In the past 10 years, many reports have focussed on the prognostic value of melanoma-associated-markers of differentiation (MAMs),^75,90,91^ such as Tyr-OH, MART-1 (Melan A/MLANA), B4GALNT1 (beta-1,4-N acetyl-galactosaminyl transferase 1), MAGE-3 (melanoma-antigen -family A3), p97 (melanotransferin/MFI2), MCAM (MUC 18/CD146, MelCAM), TRP-1/TRP-2 (tyrosinase-related protein 1 and 2), MSCP (melanoma chondroitin sulphate proteoglycan and ABCB5 (ATP-binding cassette transporter 5; Table 1).^32,73–80,83,86,88,90–99^ To date, different studies employed multi-marker assays to improve sensitivity and specificity of these procedures.^74,75,77–79,83,88–92,95^

Our research group also performed a highly specific and sensitive multi-marker RT-PCR assay focussed on Tyr-OH, MART-1, MAGE-3, MCAM/MUC18/CD146, MCAM and p97 expression levels.^85^

These MAMs were selected for their specificity and selectivity for melanoma cells, for the expression frequency in melanoma and for their high rate of detection in the RT-PCR assay.^74,75,77–79,83,85,89–92,95,96,100^ In a first study, we enrolled prospectively 100 patients (American Joint Committee on Cancer (AJCC) stages I–IV) affected by primary cutaneous melanoma or by distant metastases in cases of occult primary melanomas to analyse the co-expression of these markers in blood samples and confirm the reliability of the method.

We could validate that, among the investigated markers, Tyr-OH was the most frequent one in blood (15%), followed by MCAM/MUC18/CD146 (11%), MART-1 (5%), MAGE-3 (4%) and p97 (2%). In addition, Tyr-OH mRNA, alone or co-expressed with MART-1 or with p97, was not significantly associated with any AJCC stage. On the other hand, MART-1 and p97 were always co-expressed with Tyr-OH. MAGE-3 was detected alone or in combination with other MAMs without any significant association with AJCC stage. Our data do not confirm the report by Reynolds *et al.*^90^ that found MAGE-3 less frequently in advanced than in early stages. MAMs investigated in our series, namely, Tyr-OH, MART-, MAGE-3 and p97, resulted as not statistically related to any particular AJCC stage in contrast with the data reported on melanoma cell lines. Obviously, detection of melanoma biomarkers is hampered by the paucity of tumour material available in blood-based experiments, compared with *in vitro* growth cell lines.

Multi-marker RT-PCR assay of MAMs resulted as not so representative of the *in vivo* conditions and may give false interpretations of the gene expression. Loss of antigens—a possible event during disease progression—could occur and characterize CMCs, thus explaining our observation.

We highlighted that, when we reanalysed the patients expressing at least once MCAM/MUC18/CD146 either alone or co-expressed with other MAMs, it maintained its statistical significance with advanced stages.^85,101^

More recently, several groups developed multi-marker qRT-PCR, showing that levels of gene expression are associated with CMCs in patient blood and correlate to AJCC stage, survival and disease recurrences.^34,39,80,85,102^ In particular, qRT-PCR assays show an accurate and less laborious approach to molecular diagnosis, allowing a rapid and reproducible analysis.

Reid *et al.*^103^ in 2013 developed a multi-marker qRT-PCR for some melanocytic marker tumour-associated genes such as MCAM/MUC18/CD146, PAX3d, MART-1/MLANA, TGF*β* and a stem cell marker ABCB5. They documented that melanoma patients expressed significantly one (92 *versus* 59%) or more markers (86 *versus* 17%) than healthy donor controls. In particular, MART-1/MLANA and ABCB5 are more likely expressed in advanced AJCC stages (III–IV) and co-expression or initial expression of these markers seem to be associated with higher risk of recurrence. MCAM/MUC18/CD146 was significantly more common in non-surgically treated advanced melanoma patients with a negative outcome than in those with a positive outcome (43 *versus* 9%), reasonably due to an ineffective eradication of CMCs.

They then concluded that MART-1/ MLANA and ABCB5 are helpful in following high-risk melanoma patients confirming that MCAM/MUC18/CD146 expression is associated with poorer outcome in stage IV disease. Recently, Venditelli *et al.*^97^ improved the qRT-PCR multi-marker assay by analysing six markers, including also the markers already described by Reid *et al.*^49,103–105^ They highlighted the co-expression of PAX3d, TGF*β* and MTIFm without confirming the data by Reid *et al*.^103^ about the role of ABCB5 and MCAM/MUC18/CD146.

As reported by Mel,^39^ ‘a plethora of studies have focussed on identifying markers with sufficient specificity to accurately predict melanoma progression. Although many of these markers were identified using primary tissue or melanoma cell lines, they have been used for the multitude of CMC studies conducted until now'.^74,75,77–79,83,85,89–92,95,96,100^

Although molecular monitoring of whole-blood melanoma circulating RNA transcripts is still under debate, additional markers are required in order to understand better the diagnostic and prognostic significance of CMCs. In particular, the ability to isolate and measure CMCs represents a potentially powerful method to monitor patients with minimal morbidity and—more specifically—for an accurate diagnosis and prognosis.

### Selection, isolation and enrichment of CMCs

The rarity of CMCs in the blood stream (1–3 CMCs/~5 billion blood cells) and the lack of a standardized technology to isolate every CMC will require further efforts and technological advancements. Therefore, specific isolation and enrichment of CMC population from a clinical sample is necessary before proceeding with biological and genetic characterization of these cells. Once collected, CMCs can be subjected to molecular biology, immuno-cytochemical and cytometric approaches. Isolation of CMC based on cell size-capture technology or cell density such as filtration, density gradient centrifugation and separation from red blood cells or leukocytes requires close attention. Effectively, these strategies, which isolate CMCs and avoid the leukocyte contamination, have suggested that these cells may span a wide range of cell sizes.^106,107^ Also CMCs' isolation by analysing marker protein antigens seems complicated by the heterogeneity of the cell surface. Furthermore, CMC can acquire new genetic mutations from the tumour of origin and can be induced to phenotypic drift. This contributes to cellular heterogeneity accounting for the high variation among markers expressed by these cells.^108–111^ Some recent studies examined cytokines and phenotype profiles of freshly patient-derived human skin melanoma cells or freshly surgically excised lymph node melanoma. A great number of antigens was identified that now require validation in a larger cohort of patients and need to be explained.

In tumours as carcinomas, immuno-magnetic enrichment is the most commonly used technique for CTC collection. Immuno-magnetic antibodies against EpCAM have been used to target CTCs, followed by magnetic separation and optical analysis to isolate and reliably detect CTCs (CellSearch Circulating Tumor Cell, Janssen Diagnostic, LLC, Raritan, NJ, USA). The method that uses an antigen expressed by the tumour cells as a means to capture and isolate the aforementioned cells is referred to as ‘positive’ selection. Melanoma does not express the classical epithelial cell surface marker EpCAM, which has formed its basis for most CTC isolation,^112^ and is the only Food and Drug Administration-approved platform for the prognosis of this kind of carcinoma. Melanocytes origin from neural crest and have been associated with some specific melanoma cell surface epitopes, such as MCAM/MUC18/CD146 and MSCP/NG2 (melanoma-associated Chondroitin Sulphate) and with stem cell markers, such as ABCB5 (ATP-binging cassette subfamily member B) and CD 271.^20,32,88,96,97,113–115^

Indeed, given the difficulty of isolating whole CMCs, MCAM/MUC18/MelCAM/CD146 has been proposed as a substitute for EpCAM in immuno-targeting and separation of melanoma cells using positive selection. An alternative to the ‘positive selection’ CMC strategy is the ‘negative’ selection in which cells of interest are enriched through the depletion of unwanted cells. Negative selection is good for isolating cells with poorly characterized immuno-phenotype, such as CMCs. Figure 2 shows the main current methods for the detection of CTCs.

The presence of CTCs was identified as an independent prognostic marker in a number of metastatic cancers.^17–22,114^ The number of CTCs has been shown as indicative of the length of PFS and OS.^18,23,24^ For melanoma, few studies have explained in detail the prognostic value of CMCs. Two studies^20,114^ have shown that the number of CMCs is prognostic of OS, with >2 CMCs per 7.5 ml of blood associated with shorter survival. Both studies have been performed by using the Cell-Search Melanoma Kit (Veridex platform), which identifies double MCAM/MUC18/CD146-MCSP positive, double CD45-CD34 negative cells as CMCs.^18,23,24,116^ In particular, Khoja *et al.*^20^ indicated that 26% of metastatic melanoma patients have 2× CMCs at baseline and that median OS was shorter than those with <2 CMCs (2.6 *versus* 7.2 months, log-rank *P*<0.009). The employ of multi-marker approach has shown that the decrease in CMCs after therapy initiation is associated with response to treatment and prolonged OS in vemurafenib (anti-BRAFV600E mutation targeted therapy) treated patients. It should be noted that, the cutoff of 2 CMCs per 7.5 ml of blood is an independent prognostic biomarker. In contrast with this last data, Klinac *et al.*^17^ did not define a baseline CMC number (prior to treatment) as prognostic of OS or disease -free-survival.^17^ However, the level of circulating antigen-expressing tumour cells is still unknown, mostly due to the limited possibility to use the current technologies: CMCs are detectable in only 40% of patients with advanced melanoma. Furthermore, CMC characterization with additional markers would be added to the standard Cell-Search Melanoma Kit (ABCB5, CD 271)^17^ improving molecular subtyping and possibly tailoring the treatments.

## MCAM/MUC18/CD146/MelCAM/CD146

### Prognostic biomarker and innovative antitumoural strategy in melanoma

**Figure 3:**
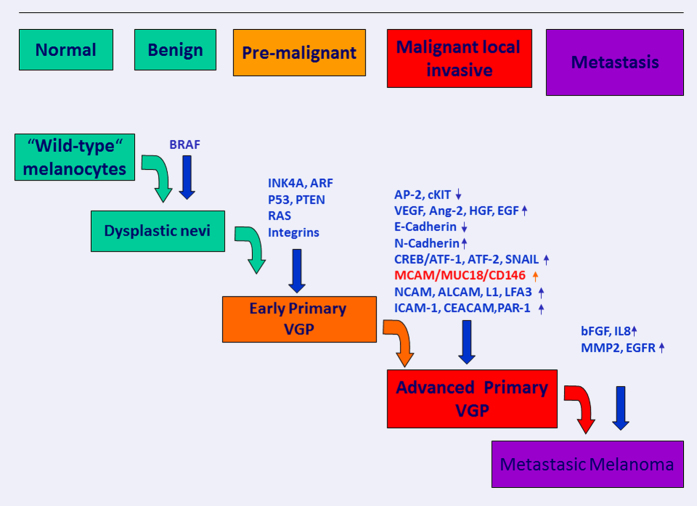
MCAM/MUC 18/CD146 input and involvement in melanoma development.

**Figure 4:**
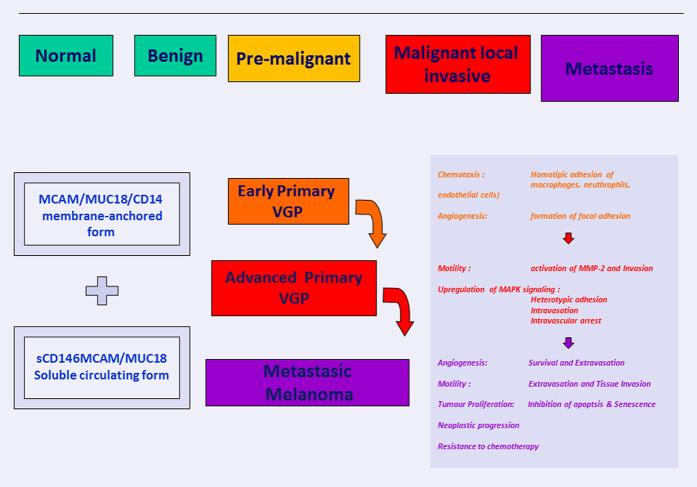
MCAM/MUC18/CD146 membrane-anchored form and sCD146/MCAM/MUC18 soluble circulating forms: biological role and activity on melanoma growth, angiogenesis and metastatic progression.

MCAM/MUC18/CD146, a melanoma cell adhesion molecule, is recently obtaining more attention as a novel biomarker for disease progression and poor outcome in patients affected by melanoma.^117–119^ Also cited as CD146, A32 antigen or S-Endo-1, it belongs to the immunoglobulin superfamily being mainly expressed at the intercellular junction of endothelial cells where it interacts directly with VEGFR-2.^98,120,121^ Originally identified in melanoma but not in normal tissue, it is now being investigated in development, signal transduction, cell migration, mesenchymal stem cells differentiation, angiogenesis and immune response.^99^ Many reports indicate that MCAM/MUC18/CD146 correlates with tumour thickness and metastatic potential of human melanoma cells in mice and in humans.^99,122–127^ As an endothelial antigen, MCAM/MUC18/CD146 affects angiogenesis promoting neoplastic progression from local invasive to metastatic disease by upregulating MMP-2 metalloproteinase and through cell interaction among extracellular matrix and vascular endothelial.^128^ Mills *et al.*^129^ studied the effect of a fully humanized anti-MCAM/MUC18/CD146 antibody (ABX-MA1) on tumour growth, angiogenesis and metastasis of human melanoma. ABX-MA1 treatment of melanoma cells was able to inhibit the promoter and collagenase activity of MMP-2 (Figures 3 and 4). Reduced MMP-2 expression was observed in implanted tumours *in vivo.*^129,130^

Moreover, as already described above, we reported that MCAM/MCAM/MUC18/CD146—either alone or co-expressed with other MAMs—maintained its statistical significance with advanced stages. The presence of MCAM/MUC18/CD146 increases the possibility of being in advanced AJCC III–IV stages and have a higher incidence of recurrences.^86,131^

All these findings strongly support a reliable role of MCAM/MUC18/CD146 in melanoma progression. Thus we decided to extend our analysis to a larger series of patients exploring circulating MCAM/MUC18/CD146 expression by RT-PCR assay on serial blood samples obtained during the clinical course of the disease.

Our investigation^132^ emphasized a correspondence among MCAM/MUC18/CD146 mRNA blood level, detection and degree of expression of this marker on the corresponding primary melanoma tissue, tumour thickness, AJCC stages and clinical outcome. We showed that MCAM/MUC18/CD146 RT-PCR assay for CMCs correlated with melanoma diagnosis and progression of the disease. Either if already detectable from the beginning or subsequently acquired during the course of the disease, MCAM/MUC18/CD146 was significantly associated with poor prognosis and death.

Differently from Reid *et al.*,^103^ we detected MCAM/MUC18/CD14 even in early AJCC stages, but surprisingly, the patients who lost this marker are still clinically disease free. On the contrary, patients affected at an early stage (AJCC stage IIB) who later acquired a persisting MCAM/MUC18/CD146 status, unfortunately then suffered from disease progression. The comparison of the clinical outcome between early AJCC stages patients sharing fleeting expression, and patients who later acquired a persisting expression, resulted statistically significant. Considering patients in advanced stages, we emphasized a statistically significant difference comparing clinical course and outcome of MCAM/MUC18/CD146-positive patients to those who never expressed this biomarker, with good outcome or stable disease. Immune surveillance or haemodynamic forces can somehow limit the different behaviour and progression.^133^ Therefore, transient CMCs expressing MCAM/MUC18/CD146—either related to the tumour burden or spread after the surgical excision—should be interpreted as limited survival early micrometastases with short half-life and consequent absence of clinical proliferating activity. While a persisting or later achieved MCAM/MUC18/CD146 detection could indicate a mature metastatic proliferative behaviour, capable of spreading into the surrounding tissue through the degrading basement membrane and extracellular matrix.^3,6,133^

In contrast with the *data* of Reid *et al.*,^103^ Venditelli *et al.*^97^ do not confirm a significant correlation of MCAM/MUC18/CD146 and ABCB5 transcripts with both Breslow classes and staging: ABCB5 does not represent a useful molecular marker for melanoma diagnosis or for melanoma targeted therapy. Moreover, in contrast with our data,^131,132^ they describe high MCAM baseline expression due to its endothelial component, constitutively expressed. Regarding this aspect, we have to focus on the two MCAM/MUC18/CD146 isoforms,^134,135^ a Long and a Short variant, different from each other for the 3’ cytoplasmic tail that may or not contain 34 amino-acid residues encoded by exon 15. This alternative splicing generates the Short isoform by direct junction of exon 14 to exon 16, with the excision of exon 15. The Short isoform is widely expressed by endothelial cells, rather than the Long isoform that seems expressed preferentially by melanoma primary culture cells. An interesting commentary on the publication of Reid *et al*.^103^ has been previously reported.^136^ The authors underline that MCAM transcripts, analysed by qRT-PCR, may fluctuate significantly in the healthy population. They suppose that the elevated copy number, also present in normal individuals, could be related to one of the two MCAM/MUC18/CD146 isoforms. Therefore, they hypothesize a possible MCAM transcript overestimation mainly due to Short isoform detection. They explain this feature by considering intercellular interactions between melanoma cells and vascular endothelium, particularly during metastatic process. Furthermore, they suggest a particularly accurate design of primers and probes in order to minimize the effect of endothelial contamination.^136^ According to this last consideration, we describe our preliminary pilot study performed on CMCs isolated and enriched from patients affected by melanoma at least by AJCC stage Ib, by targeting the MCAM/MUC18/CD146 antigen with immune-magnetic beads coated with antibody against MCAM/MUC18/CD146 antigen. The expression study of Long, Short isoforms and extracellular domain of MCAM/MUC18/CD146, performed by home-made designing specific primers, nicely documented the co-expression of both isoforms on enriched cells isolated from melanoma patients with respect to healthy subjects only carrying the Short variant. In our view, the most interesting emerging data are obtained by using primers mapping at the 5’ upstream of the transcript (NM_006500-3332 bp).^74^ Effectively, the persistent expression or achievement of this specific molecular transcript seems to characterize advanced melanoma status, also analysing selected CD146-positive cells. The transcript, corresponding to the first extracellular domain of the antigen, is not detected in circulating enriched cells selected from healthy donors, even if expressing the Short isoform. Obviously, an accurate CMC enrichment need to be assessed and these preliminary data need to be confirmed. The group of Blot-Chabaud^137,138^ identified, in addition to the membrane-anchored form of MCAM/MUC18/CD146, a soluble form of CD146 (sCD146/MCAM/MUC18),^139–141^ which is mainly generated by the proteolytic cleavage of the membrane form through metalloproteases.^141^ Interestingly, the sCD146/MCAM/MUC18 concentrations increased in several diseases, in particular in tumours.^142^ Moreover, sCD146/MCAM/MUC18 constitutes an active factor having a major role in angiogenesis.^143^ They documented that this effect was mediated through the binding of sCD146/MCAM/MUC18 on the p80 isoform of angiomotin.^120^ This protein is not only detectable on the vasculature of ischemic tissues^143^ but also on many tumour cells.^144–149^ These findings suggest that MCAM/MUC18/CD146-positive tumours could secrete soluble CD146 that, in turn, would be responsible for their growth and vascularization. In particular, in this study, sCD146/MCAM/MUC18 secreted by CD146-positive tumours does not only display effects on tumour angiogenesis but also on tumour growth and survival. Thus sCD146/MCAM/MUC18 induces the expression of either proteins involved in tumour proliferation and invasion or proteins inhibiting apoptosis and senescence. The decision to focus the attention on the circulating form of the protein could be extremely powerful to target sCD146/MCAM/MUC18 with monoclonal antibody capable of neutralizing its effects without affecting the membrane MCAM/MUC18/CD146. This approach could constitute an innovative antitumoural strategy. These data demonstrate that the active powerful action on angiogenesis, tumour growth and disease progression, is exerted by the extracellular portion of the protein. Taken together, MCAM/MUC18/CD146 molecular expression analysis, sequential monitoring of the transcripts and detection of the soluble form could help to investigate or follow the melanoma remission or progression even in apparent disease-free status. MCAM/MUC18/CD146 behaves as a ‘molecular warning of progression’ and its targeting could constitute an innovative therapeutic strategy for the CD146-positive melanoma.

## Figures and Tables

**Table 1:** Melanoma biomarkers utilized for detection of CMCs

*Melanoma Biomarkers*	*References*
Tyrosinase (Tyr-OH)	Smith *et al.*^73^; Hoon *et al.*^74^; Curry *et al.*^75^; Brownbridge *et al.*^95^
Melan A/MART 1	Hoon *et al.*^74^; Curry *et al.*^75^; Tsukamoto *et al.*^77^; Schittek *et al*.^83^; Brownbridge *et al.*^95^
MCAM/MUC18/CD146	Hoon *et al.*^74^; Curry *et al.*^75^; Lehmann *et al.*^98^; Melnikova and Bar-Eli ^99^
MAGE 3	Hoon *et al.*^74^; Curry *et al.*^75^; Gaugler *et al.*^76^
P97/Melanotransferin/MFI2	Rose *et al.*^80^; Hoon *et al.*^74^; Curry *et al.*^75^
TRP-1/TRP-2	Jiménez-Cervantes *et al.*^91^; Sarantou *et al*.^93^
B4GALNT1 (beta-1,4-*N* acetyl-galactosaminyl transferase 1)	Kuo *et al.*^86^; Sarantou *et al*.^93^
MCSP (melanoma chondroitin sulphate proteoglycan)	Yang *et al.*^88^
ABCB5 (ATP-binding cassette transporter 5)	Schatton and Frank^32^; Frank *et al.*^96^; Venditelli *et al.*^97^
CD 271 (neural crest nerve growth factor receptor)	Boiko *et al*.^113^; Luo *et* *al*.^115^
